# Supplementary material for: Diabetic phenotype in mouse and humans reduces the number of microglia around β-amyloid plaques
Source: Mol Neurodegener. 2020 Nov 10;15:66. doi: 10.1186/s13024-020-00415-2 (PMC7653710; doi:10.1186/s13024-020-00415-2)
Supplement: Supplementary file 1 — Additional file 1: Supplementary Figure S1. a-c. Assessment of soluble Aβ42 and Aβ40 levels as well as Aβ42 and Aβ40 ratio of hippocampal samples from mice with A+ transgene (APPswe/PS1dE9) revealed no significant diet effect. Data are presented as mean + SEM, n = 5–6, Two-way ANOVA. d Representative immunofluorescence images of β-amyloid plaques stained with X-34 (blue) surrounded by 22C11-positive dystrophic neurites (red), and Iba1-positive microglia (green) from A+Tw and A+T+ mice from both STD and TWD groups. Scale bar 10 μm. e Counting of 22C11-positive neurites revealed no diet nor genotype effect. f Quantification of 22C11-positive area (pixels) around β-amyloid plaques revealed a statistically significant genotype x diet interaction (p < 0.001). TWD significantly increased the 22C11-positive area (p = 0.001), while T+ transgene (Tau P301L) had an opposing effect on the 22C11-positive area depending on the diet. d-f; results are shown as mean + SEM, n = 5–6 mice/group, Two-Way ANOVA. g A representative Western blot image of hippocampal lysates and quantification phospho-Tau and total Tau levels. Phosphorylated protein levels were normalized to their respective total protein levels in cell lysates and total protein levels were normalized to β-actin. All results are shown as mean + SEM, n = 5–7 mice/group, Two-way ANOVA. A+Tw = APPswe/PS1dE9, A+T+ = APPswe/PS1dE9 x Tau P301L. Supplementary Figure S2. a. Heatmap of z-score values for transcripts specifically mapping to human (h) APP, PSEN1, and MAPT, show a significant increase in APP and PSEN1 in A+Tw and A+T+ mice, and a significant increase in MAPT levels for AwT+ and A+T+ mice as compared to AwTw mice. b PCA for all samples (AwTw, AwT+, A+Tw, A+T+ STD/TWD). c PCA for samples of each genotype showing segregation due to TWD. d Number of DE genes (FDR < 0.05) for each genotype/diet combination as compared to corresponding diet AwTw mice. e Venn diagram showing overlap of DE genes between genotype/diet groups. [file 13024_2020_415_MOESM1_ESM.pdf]

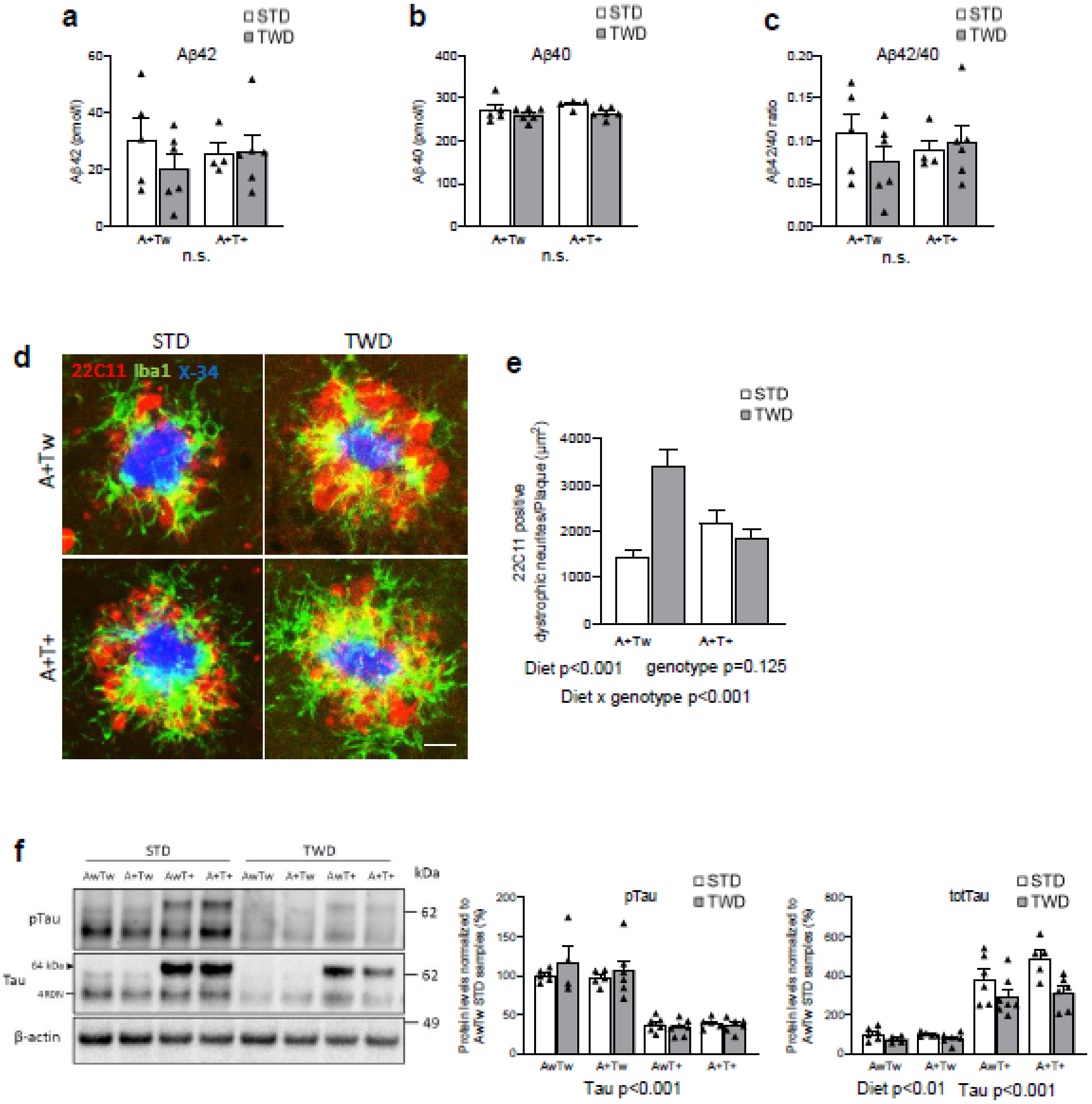

Supplementary Figure 1

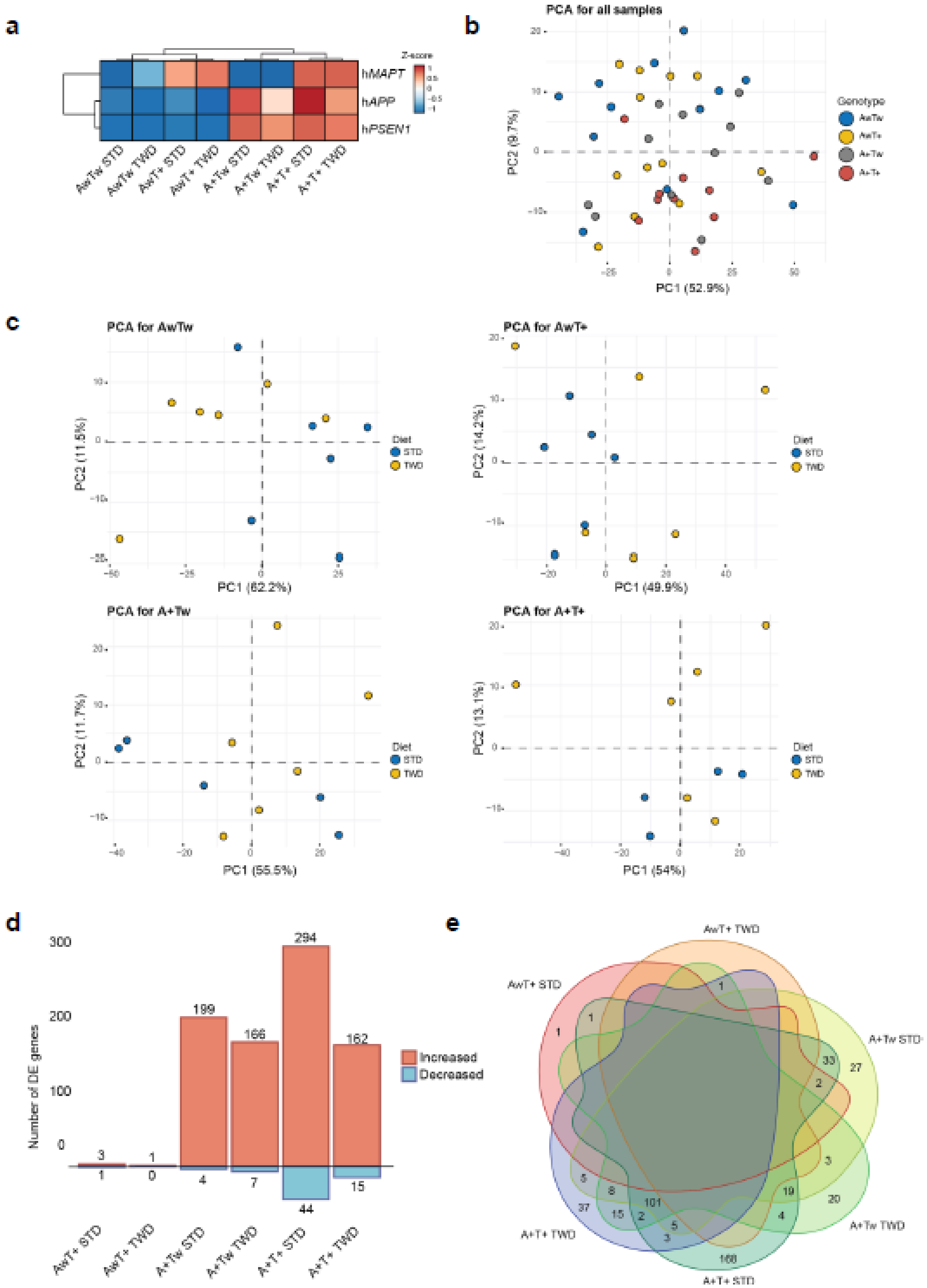

Supplement Figure 2

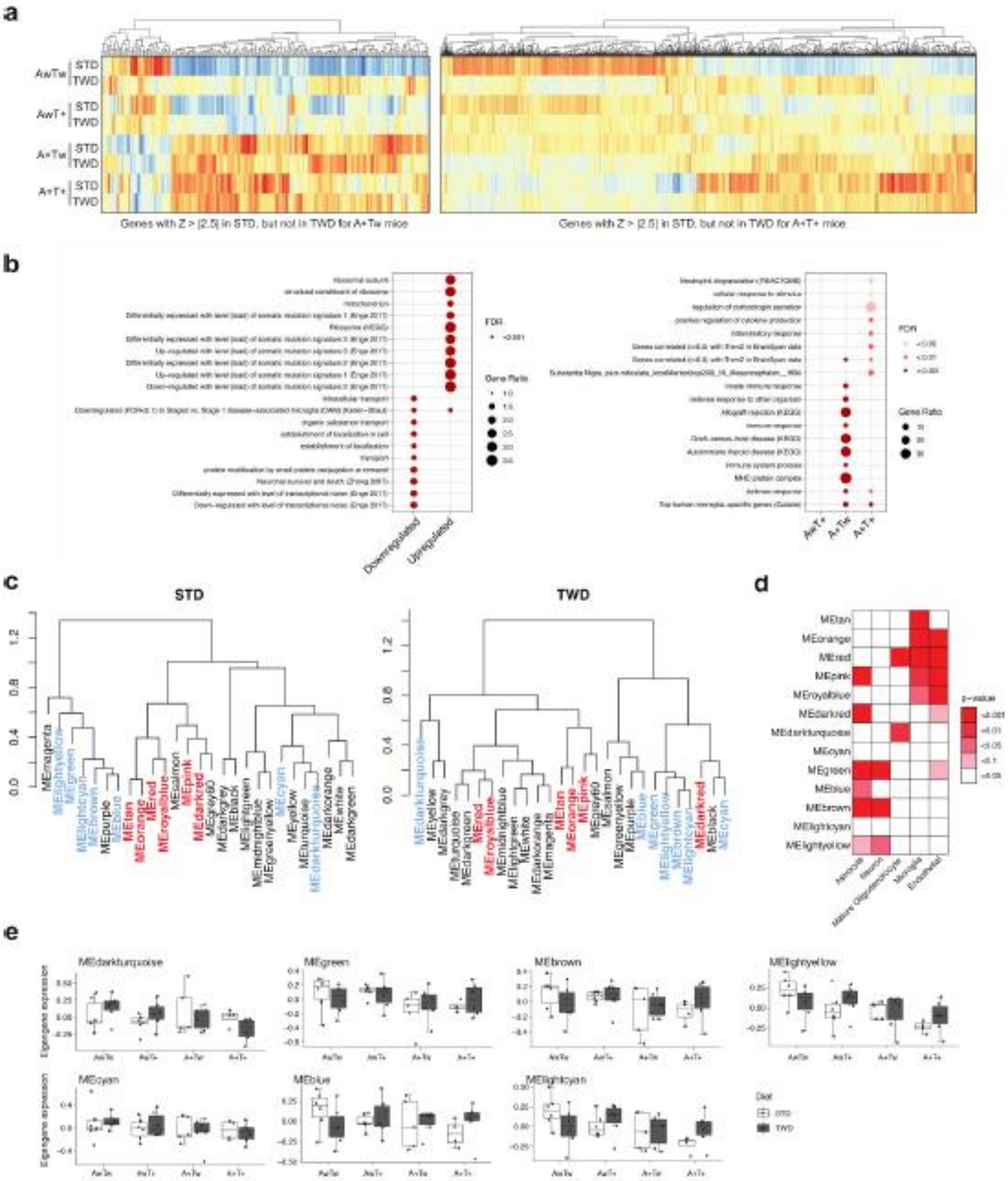

Supplement Figure 3

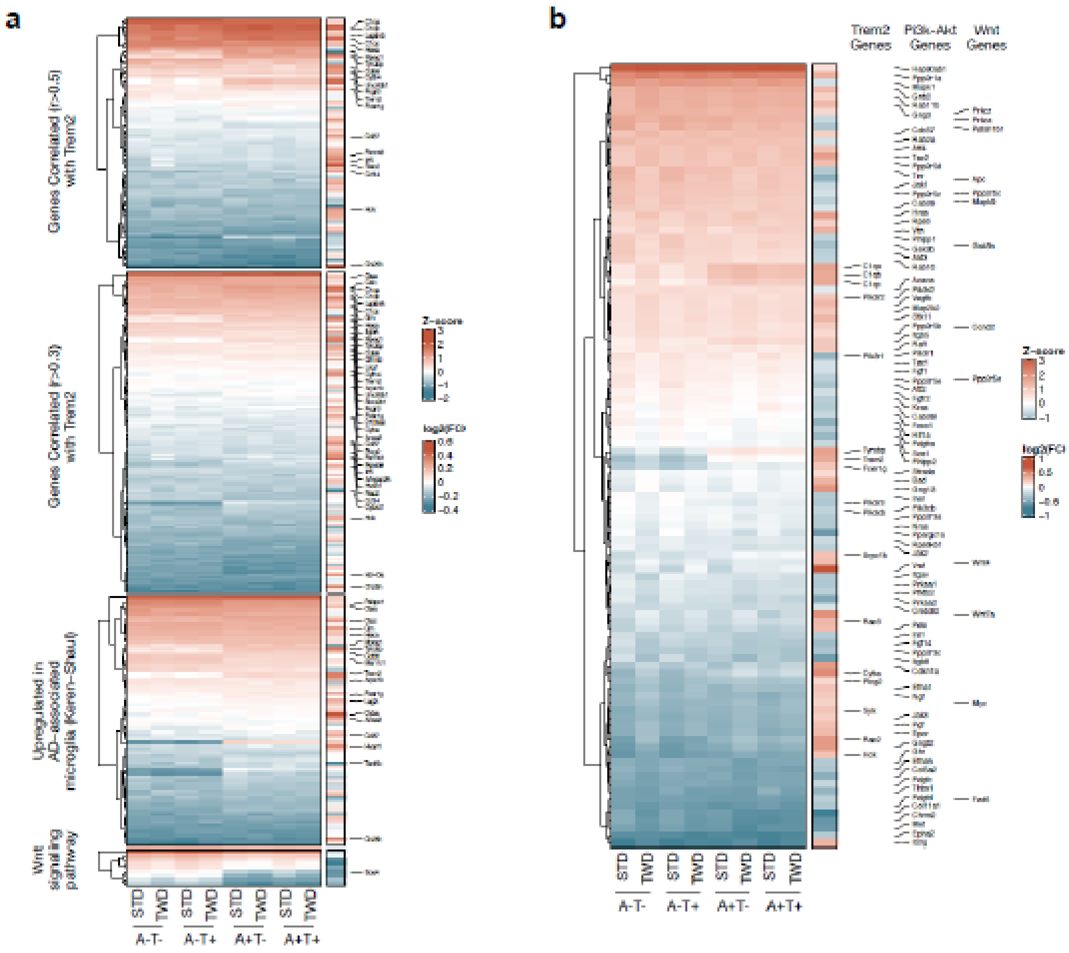

Supplementary Figure 4

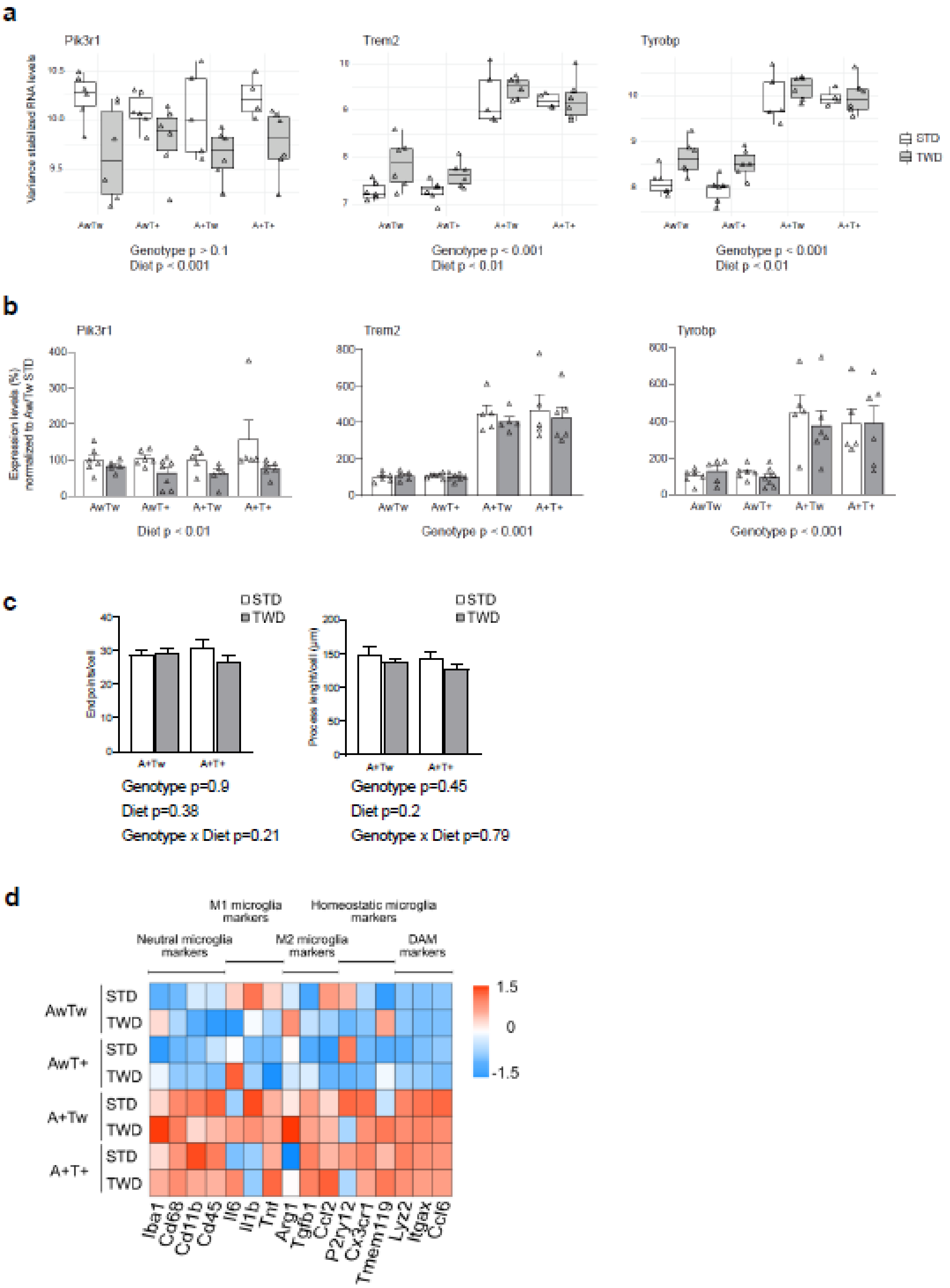

Supplement Figure 5

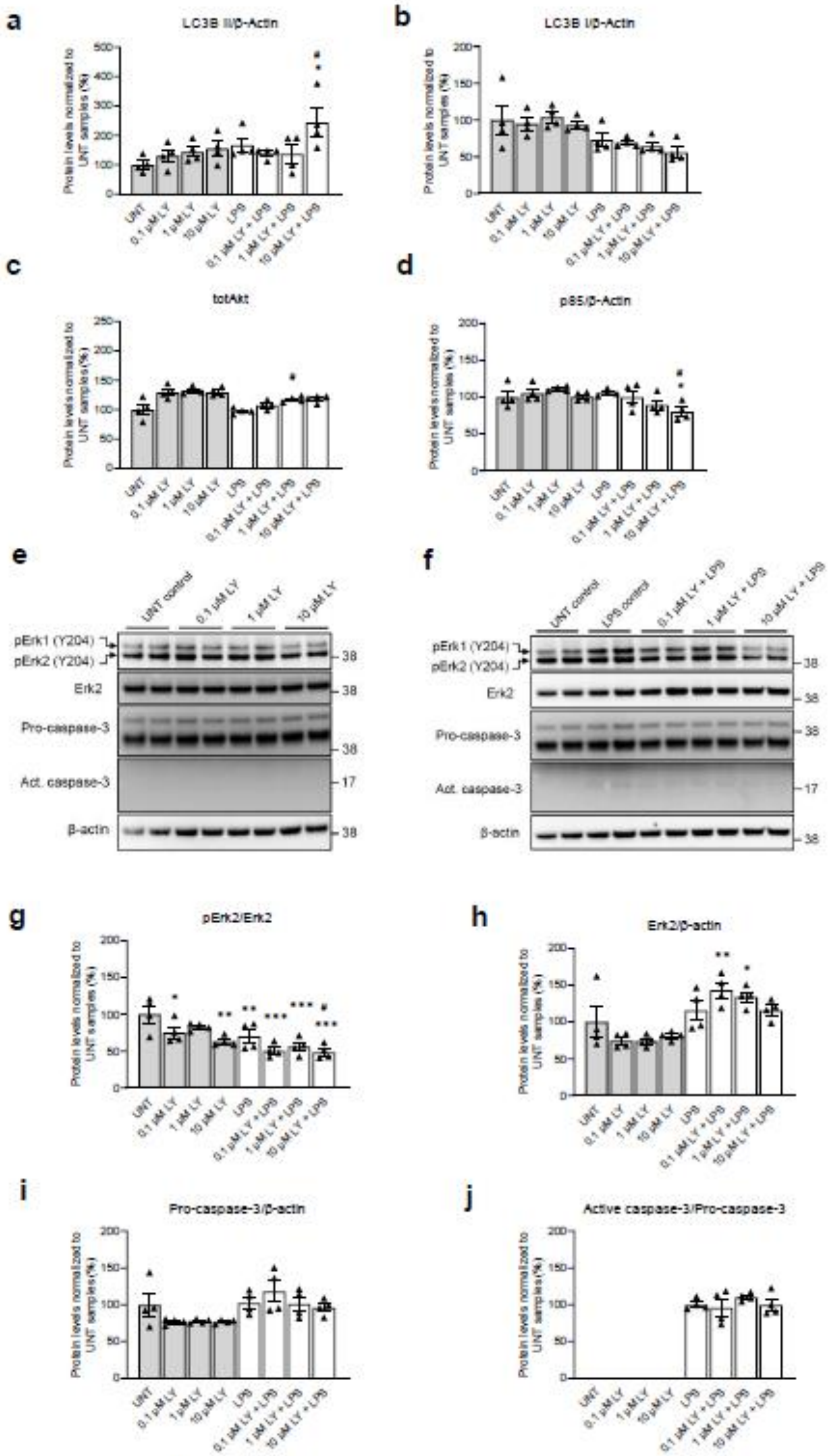

Supplement Figure 6
